# Mutation analysis of paired box 6 gene in inherited aniridia in northern China

**Published:** 2013-05-30

**Authors:** Peng Chen, Xinjie Zang, Dapeng Sun, Ye Wang, Yao Wang, Xiaowen Zhao, Mohan Zhang, Lixin Xie

**Affiliations:** State Key Laboratory Cultivation Base, Shandong Provincial Key Laboratory of Ophthalmology, Shandong Eye Institute, Shandong Academy of Medical Sciences, Qingdao, China

## Abstract

**Purpose:**

Aniridia is phenotypically and genetically heterogeneous. This study is to summarize the phenotypes and identify the underlying genetic cause of the paired box 6 (*PAX6*) gene responsible for aniridia in two three-generation Chinese families in northern China.

**Methods:**

A detailed family history and clinical data were collected from patients during an ophthalmologic examination. All exons and flanking intronic sequences of the *PAX6* gene were amplified with PCR and screened for mutation with direct DNA sequencing. Haplotyping was used to confirm the mutation sequence. Real-time PCR was used to determine the *PAX6* messenger ribonucleic acid(mRNA) level in patients with aniridia and in unaffected family members.

**Results:**

The probands and other patients in the two families were affected with aniridia accompanied with or without congenital cataract. A heterozygous *PAX6* mutation in exon 5 (c.112delC, p.Arg38GlyfsX16) was identified in FAMILY-1, which was predicted to generate a frameshift and created a premature termination codon. A heterozygous *PAX6* mutation in exon 7 (c.362C>T, p.Ser121Leu) was identified in FAMILY-2. Each mutation cosegregated with the affected individuals in the family and did not exist in unaffected family members and 200 unrelated normal controls. The *PAX6* messenger ribonucleic acid level was about 50% lower in patients with aniridia than in unaffected family members in FAMILY-1.

**Conclusions:**

The deletion mutation (c.112delC) in the *PAX6* gene was first identified in a Chinese family with aniridia, congenital progressive cataract, developmental delay, or the absence of ulna. The mutation (c.362C>T, p.Ser121Leu) in the *PAX6* gene was first identified in a patient with aniridia with congenital ptosis. We summarized the variable phenotypes among the patients, which expanded the phenotypic spectrum of aniridia in a different ethnic background.

## Introduction

Aniridia (OMIM 106210) is a rare, bilateral, congenital ocular disorder causing incomplete formation of the iris. The degree of iris hypoplasia is variable, ranging from minimal loss of iris tissue to nearly complete absence. The visual impairment caused by iris hypoplasia might be enhanced by several ocular complications, including cataract, glaucoma, and corneal clouding. In 85% of individuals with aniridia, this disorder is inherited as an autosomal dominant trait, in 13%, aniridia occurs as part of the autosomal dominant Wilms’ tumor, aniridia, genitourinary abnormalities, and mental retardation (WAGR) syndrome [1], and in the remaining 2%, aniridia occurs as part of other disorders, including Peters anomaly [2] and Gillespie syndrome [3], in either autosomal dominant or autosomal recessive inheritances.

Congenital aniridia is inherited as an autosomal dominant trait with high penetrance and variable expressivity [4,5]. The aniridia gene has been mapped on chromosome 11p13 by linkage analysis and positional cloning. The pair box 6 (*PAX6*) gene located at 11p13 has been confirmed as the major gene associated with aniridia [6-9]. PAX6 has two DNA binding domains, a bipartite paired domain (PD) and a paired-type homeodomain (HD), as well as a transactivation domain-rich proline, serine, and threonine at the COOH-terminal end. The PD and the HD, which are separated by a linker region, are the structural bases for the binding activity of the PAX6 protein.

Congenital cataract (OMIM 604307) is an opacification of the eye lens resulting in visual impairment or even blindness during infancy or early childhood [10]. According to the Human *PAX6* Allellic Variant Database [11], some *PAX6* mutations have been reported to be associated with aniridia accompanied by congenital cataract. However, identified mutations are located throughout the length of *PAX6* with limited clear evidence of genotype-phenotype correlation.

*PAX6* mutations have been reported in Chinese [12-28], and disease phenotypes vary among different *PAX6* mutations. The deletion mutation (c.112delC) in the *PAX6* gene has been reported in various ethnic backgrounds [29-34]. However, it was first identified in a Chinese family with aniridia and congenital progressive cataract. The mutation (c.362C>T) in the *PAX6* gene was first identified in patients with aniridia. The phenotype-genotype correlation, which is important in understanding the disease mechanism, remains to be further elucidated. In this study, we present the clinical and molecular genetic evaluations performed on two three-generation Chinese families with aniridia and identify a 1 bp deletion and a novel heterozygous mutation.

## Methods

### Subject recruitment and clinical examination

**Figure 1:**
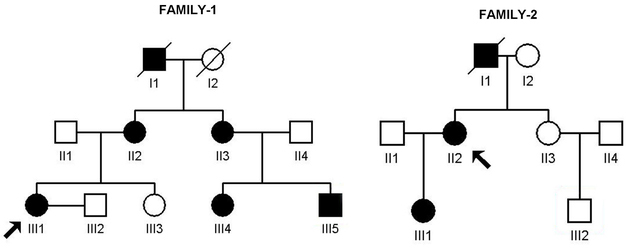
Pedigrees of the two Chinese families with autosomal dominant aniridia. Affected men and women are indicated by filled squares and circles, respectively. Normal individuals are shown as empty symbols. Deceased individuals are indicated with slashes (/). The proband is indicated with an arrow.

Two families with autosomal dominant aniridia in three successive generations were recruited at the Qingdao Eye Hospital, Qingdao, China. The two families came from Qingdao (Shandong, China).There were nine individuals in FAMILY-1(five affected and four unaffected, four male and five female). There were seven individuals in FAMILY-2(two affected and five unaffected, three male and four female). Patient II:3 in FAMILY-1 was 128 cm tall, and sheweighed 52 kg. Patient III:5 in FAMILY-1 was 105 cm tall, and he weighed 18 kg with the absence of the ulna in his left forearm. There was no family history of other systemic abnormalities in FAMILY-1and FAMILY-2. The study was performed in accordance with the Declaration of Helsinki and approved by the Ethical Review Committee of Shandong Eye Institute, and informed consent was obtained from all participants. The diagnosis was confirmed with ophthalmologic examinations, including visual acuity, slit-lamp examination, tonometer, keratometry, corneal endothelium examination, ultrasonic A/B scan, or a history of cataract extraction. Ocular photographs were taken by slit-lamp photography without pupil dilation. Sixteen individuals (seven affected and nine unaffected) from the families participated in the study (Figure 1). Two hundred subjects (27.20±7.13 years old, 117 male) from the same population without diagnostic features of aniridia were recruited to serve as normal controls. After informed consent was obtained from all participating individuals following the principles of the Declaration of Helsinki, peripheral venous blood samples were collected for genomic DNA extraction from the blood leucocytes.

### Genomic deoxyribonucleic acid preparation and molecular analysis

Venous blood (5 ml) was collected from the participants, and total human genomic DNA was isolated with the DNA isolation kit for mammalian blood (Tiangen, Beijing, China). Venous blood and genomic DNA samples were stored at −80 °C before use. Mutation screening of the *PAX6* gene (RefSeq: NM_000280.4) was performed with Sanger sequencing. Gene-specific PCR primers were designed and used to amplify individual exons and flanking intron sequences applying standard PCR amplification protocols. The PCR products were subsequently sequenced with the 3130×l Genetic Analyzer (Applied Biosystems, Foster City, CA). Primer sequences were given in Table 1.

**Table 1:** Primers used for polymerase chain reaction amplification and sequencing of *PAX6*.

**Exon**	**Forward /sequencing primer**	**Reverse primer**	**Product size (bp)**
1	AGGGAACCGTGGCTCGGC	GGGTGAGGGAAGTGGCTGC	207
2.1	TTATCTCTCACTCTCCAGCC	CTGTTGTTGCTTGAAGACCAC	178
2.2	AAACTCTCACCAGCAACTCC	GGAGACCTGTCTGAATATTGC	197
3	GGACGTATGCTGTTGAACCAC	TGAGCCCAAAGCAGCCACCA	166
4	AACAGAGCCCCATATTCGAG	AGTCCCTGTGTCCTCCCC	142
5a	CTCTACAGTAAGTTCTCATACC	GGAAGTGGACAGAAAACCAC	173
5	CTCTTCTTCCTCTTCACTCTGC	GAAATGAAGAGAGGGCGTTG	268
6	TCAAAACGTAAGCTTGTCATTG	ACAGTGGAGAGAGAGGGTGG	403
7	TGGGTGACTGTGTCTTCAGG	AATGGTTGGGAGAGTAGGGG	300
8	TTAAGACTACACCAGGCCCC	TGAAGATGTGGCATTTACTTTG	294
9–10	TTGATGCACAGTTTGGTCAAC	GTGAGAGTCAGAGCCCGGAG	602
11	AAACCTGTTTGCTCCGGG	CAATGAGGTCCGCAGGC	256
12	GGCTGTGGCTGTGTGATG	ACCAGGAGATTCTGTTTGGG	285
13	CCATGTCTGTTTCTCAAAGGG	AAGCTCAACTGTTGTGTCCC	222

Haplotyping was used to confirm the mutation sequence. PCR products of heterozygous mutants were ligated to pMD18-T vectors, and sequenced to identify the mutation. Briefly, PCR products were purified by gel extraction using gel extraction kits (Tiangen) according to the manufacturer's instructions. The purified PCR fragments were ligated into the pMD18-T vector, and the resulting plasmids were transfected by heat shock into DH5a competent *Escherichia coli* for propagation. Glycerol stocks were frozen to maintain the clones. Colonies were picked and grown overnight in 1–2 ml of Luria-Bertani broth. Plasmids were purified using the plasmid extraction kit (Tiangen). The plasmid DNA was sequenced using the 3130×l Genetic Analyzer.

The sequencing results were compared with the reference sequences in the database at the National Center for Biotechnology Information (NCBI). Mutation descriptions follow the new nomenclature system recommended by the Human Genomic Variation Society (HGVS) [35].

### Ribonucleic acid extraction and real-time polymerase chain reaction

Total RNA was prepared from venous blood (0.2 ml) of all the family members, using the RNA isolation kit for mammalian blood (Tiangen). One microgram of total RNA from each sample was reverse transcribed into cDNA, and real-time PCR was performed using the SYBR Premix Ex Taq kit (Tiangen) in accordance with the manufacturer’s instructions. The primer sequences for the *PAX6* gene were 5ʹ-TTC ACA TCT GGC TCC ATG TT-3ʹ (forward) and 5ʹ -GGG TTG CAT AGG CAG GTT AT-3ʹ (reverse). As an internal control, the glyceraldehyde-3-phosphate dehydrogenase gene was assessed with the primers 5ʹ-ATG CTG GCG CTG AGT ACG T-3ʹ (forward) and 5ʹ-AGC CCC AGC CTT CTC CAT-3ʹ (reverse).

## Results

### Clinical evaluation

**Figure 2:**
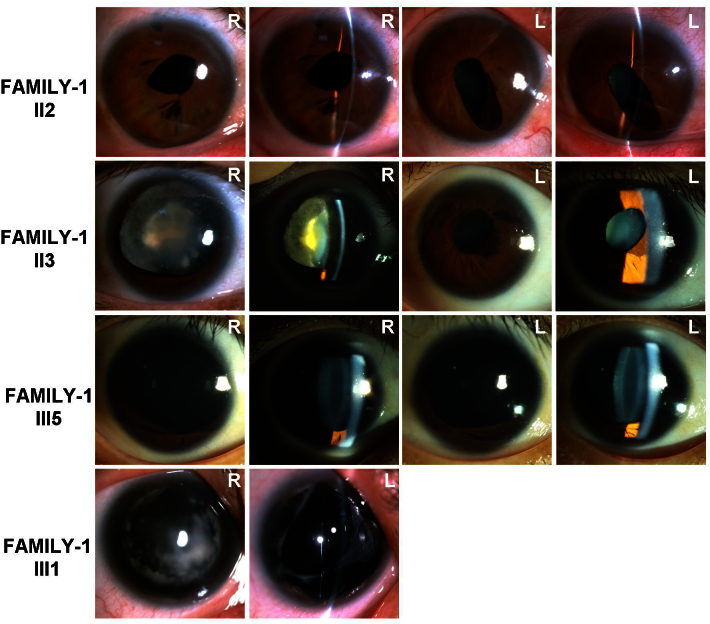
Slip-lamp photographs of the affected individuals in FAMILY-1. The phenotypes are described and summarized in Appendix 1. R: right eye, L: left eye.

We identified a three-generation family with autosomal dominant aniridia and congenital progressive cataract (Figure 1A, Figure 2). Seven eyes were categorized into complete aniridia, and three eyes were categorized into partial aniridia. The best-corrected visual acuity ranged from finger counting to 40/200. All affected patients had horizontal nystagmus. Corneal curvature ranged from 41.0 to 47.63 D (42.26±1.11 D in the minimal meridian and 44.13±2.16 D in the maximal meridian). Ectopia lentis was detected in patient II:3, but not in patient II:2, III:1, III:4, or III:5. Patient II:3 was 128 cm tall, and she weighed 52 kg. Patient III:5 was 105 cm tall, and he weighed 18 kg with the absence of the ulna in his left forearm. There was no family history of other systemic abnormalities. All clinical findings are summarized in Appendix 1.

**Figure 3:**
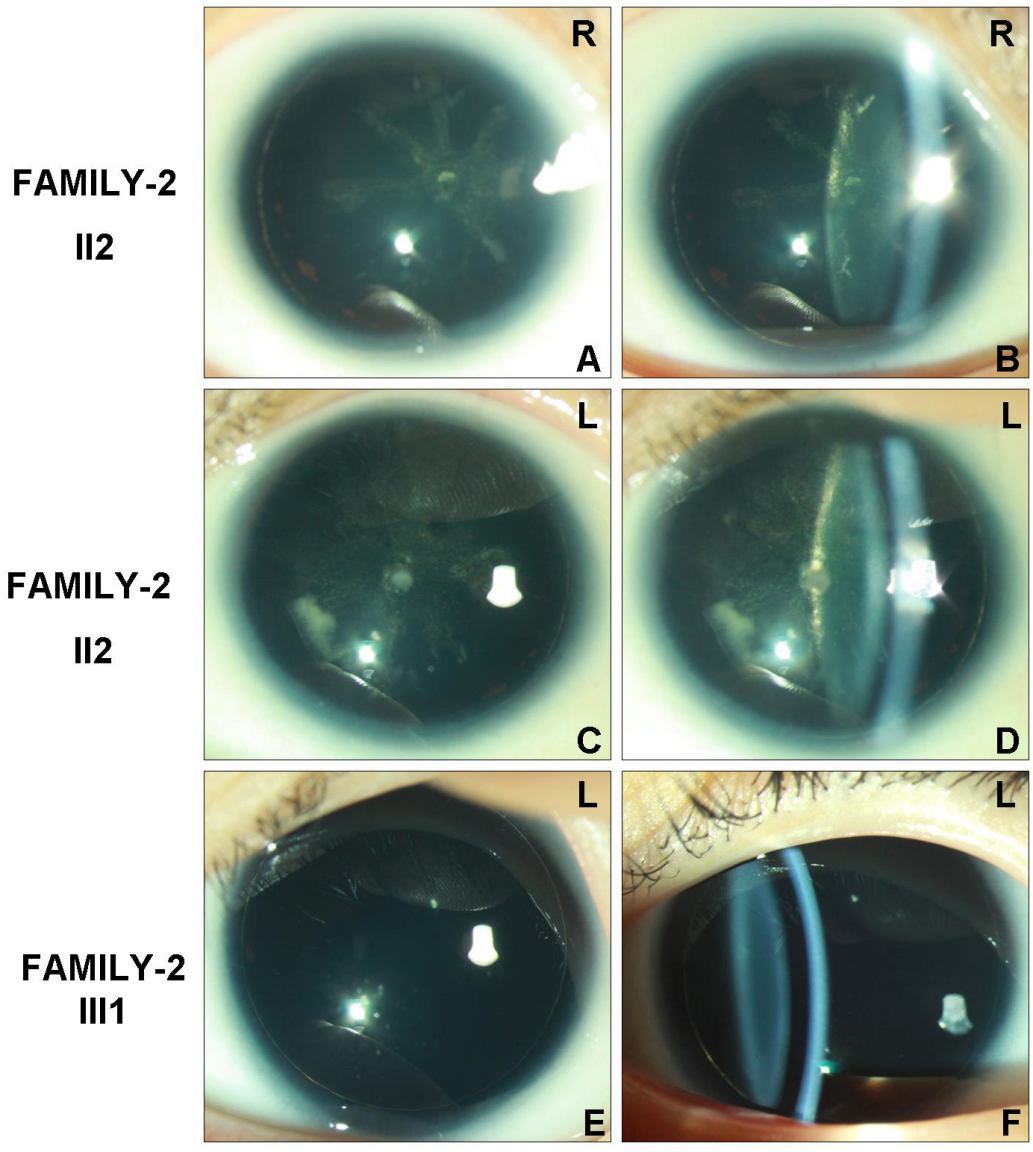
Slip-lamp photographs of the affected individuals in FAMILY-2. The phenotypes are described and summarized in Appendix 1. R: right eye, L: left eye.

We identified another three-generation family with autosomal dominant aniridia (Figure 1B, Figure 3). Bilateral total aniridia, congenital cataract, and congenital horizontal nystagmus were present in the proband (Figure 3A–D). Patient III:1 in the family had no congenital cataract (Figure 3E,F). Bilateral total aniridia, congenital horizontal nystagmus, and congenital ptosis were present in patient III:1 (Figure 3E,F). There was no family history of other systemic abnormalities. All the clinical findings are summarized in Appendix 1.

### Mutation analysis

**Figure 4:**
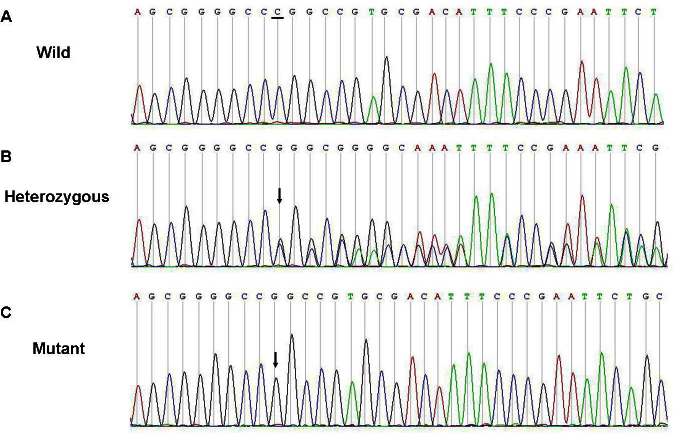
Sequence of the polymerase chain reaction product of exon 5 in paired box 6 gene in FAMILY-1. The top chromatogram represents the sequence of a normal family member (III:3). The middle chromatogram shows a reading frame shift in the proband (III:1, heterozygous), and the arrow indicates the initiation of the mutation site (beginning of overlapping peaks). The bottom chromatogram shows the sequence from the haplotyping of the mutation sequence, and the arrow indicates the location of the mutation.

Direct sequencing of *PAX6* in all affected patients in FAMILY-1 revealed a heterozygous 1 bp deletion (c.112delC) within the paired domain in exon 5 (Figure 4). The c.112delC generated a frameshift and a premature termination 16 codons downstream.

**Figure 5:**
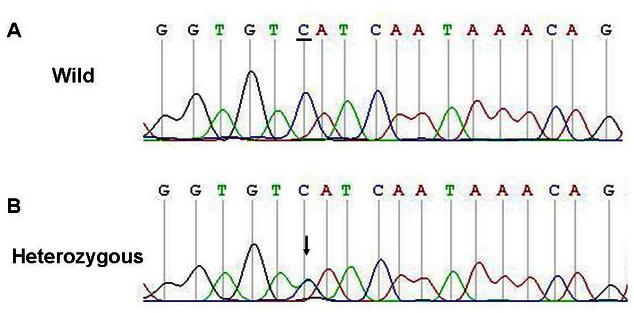
Sequence of the polymerase chain reaction product of exon 7 in paired box 6 gene in FAMILY-2. The top chromatogram shows the sequence of a normal family member (II:1). The bottom chromatogram shows the sequence from the proband (II:2, heterozygous), and the arrow indicates the location of the mutation.

A heterozygous mutation (c.362C>T) was detected in exon 7 of the two affected individuals in FAMILY-2 (Figure 5). The mutation resulted in the substitution of a serine codon for a leucine codon (p.Ser121Leu). The c.112delC and c.362C>T mutations were not detected in the unaffected members of the families or in any of the 200 normal Chinese Han controls from the same ethnic background who were analyzed.

### Computational analysis

**Figure 6:**
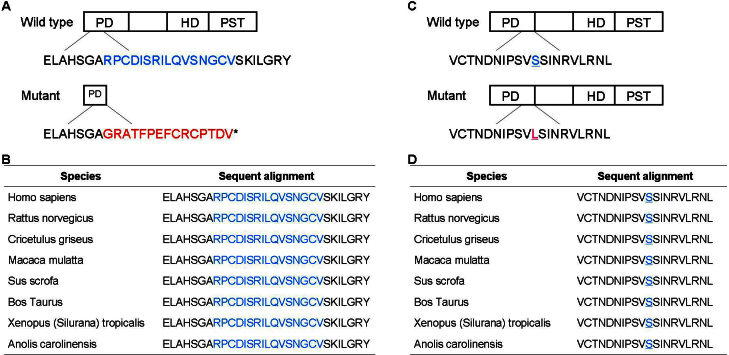
The mutational regions in the paired box 6 protein are highly conserved among different species. **A**: Diagrams of the wild-type and mutant paired box 6 (PAX6) proteins in FAMILY-1. The upper panel shows a human wild-type PAX6 protein. The lower panel shows the mutant in FAMILY-1. The c.112delC generates a frameshift and a premature termination 16 codons downstream (p. Arg38GlyfsX16). The peptide in blue encoded by exon 5 of the wild-type is replaced by the peptide in red in the mutant. **B**: Multiple alignments of Arg38-Val53 of human PAX6 protein from different species revealed 100% identity, which suggested that it was highly conserved during evolution. **C**: Diagrams of the wild-type and mutant PAX6 proteins in FAMILY-2. The upper panel shows a human wild-type PAX6 protein. The lower panel shows the mutant in FAMILY-2. The peptide in blue encoded by exon 7 of the wild-type is replaced by the peptide in red in the mutant. **D**: Multiple alignments of Val111-Leu130 of human PAX6 protein from different species revealed 100% identity, which suggested that it was highly conserved during evolution.

The c.112delC generated a frameshift and a premature termination 16 codons downstream (p.Arg38GlyfsX16; Figure 6A). Multiple alignments of Arg38-Val53 of the human PAX6 protein (Homo sapiens, NP_000271.1) from different species revealed 100% identity, which suggested that it was highly conserved during evolution (Figure 6B).

The c.362C>T generated a missense mutation (p.Ser121Leu; Figure 6C). Multiple alignments of Ser121 of the human PAX6 protein from different species revealed 100% identity, which suggested that it was highly conserved during evolution (Figure 6D). The Sorting Intolerant From Tolerant (SIFT) tool analysis revealed a score of <0.05 and predicted that the replaced amino acid was “damaging” to protein function. The Polymorphism Phenotype (PolyPhen) tool analysis revealed that the replaced amino acid was “probably damaging” to protein function.

The various species included *Rattus norvegicus* (NP_037133.1), *Bos taurus* (NP_001035735.1), *Macaca mulatta* (NP_001253186), *Xenopus* *(Silurana) tropicalis* (NP_001006763.1), *Sus scrofa* (NP_001231102.1), *Cricetulus griseus* (XP_003497614.1), and *Anolis carolinensis* (XP_003214735.1).

### Paired box 6 gene messenger ribonucleic acid expression in patients with aniridia in FAMILY-1

**Figure 7:**
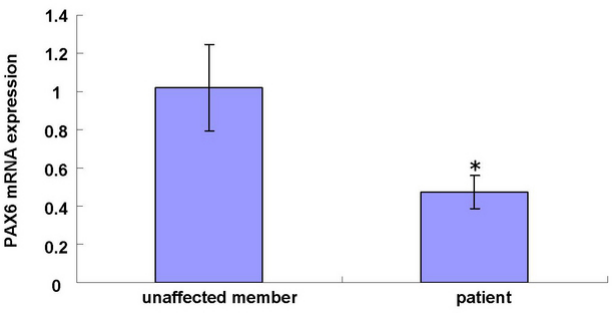
Confirmation of the change in the paired box 6 gene messenger ribonucleic acid expression in patients with aniridia and unaffected members of the family using real-time polymerase chain reaction. The paired box 6 (*PAX6*) gene messenger ribonucleic acid (mRNA) level (normalized to glyceraldehyde-3-phosphate dehydrogenase) was about 50% lower in patients with aniridia than in unaffected members (*p<0.01).

To confirm the change in *PAX6* messenger ribonucleic acid (mRNA) expression in patients with aniridia and unaffected family members in FAMILY-1, total RNA was prepared from venous blood, and reverse transcription was followed by real-time PCR. The *PAX6* mRNA level (normalized to glyceraldehyde-3-phosphate dehydrogenase) was about 50% lower in patients with aniridia than in unaffected family members (*p<0.01; Figure 7). The differences between the patients and unaffected members were tested using Student *t* tests.

## Discussion

In the present study, the identified mutation (c.112delC) generated a frameshift and a premature termination 16 codons downstream (p.Arg38GlyfsX16). Nonsense-mediated decay (NMD) is the process by which mRNAs containing premature termination codons (PTCs) are degraded before the supposed truncated proteins are produced [36,37]. The mutation in *PAX6* was predicted to result in a transcript recognized by the nonsense-mediated mRNA decay system [38] leading to a half reduction of the full-length PAX6 protein. This model was verified in our testing. The *PAX6* mRNA level was about 50% lower in patients with aniridia than in unaffected family members in FAMILY-1 (Figure 7). This result conforms to genotype-phenotype correlation analysis, suggesting that mutations that introduce a PTC into the open reading frame usually result in the aniridia phenotype [39].

The c.112delC mutation had been previously reported in sporadic patients. It is associated with isolated aniridia, which has been found in a Dutch population [31]. This mutation is also associated with aniridia accompanied by nystagmus, cataract, glaucoma, myopia, foveal hypoplasia, and attention deficit hyperactivity disorder in populations in Saudi Arabia and Turkey [33,34]. Our study first identified the *PAX6* c.112delC mutation in a large Chinese pedigree.

The c.362 C>T mutation, rather than a rare polymorphism in the normal population, is the causative mutation in the family. The novel characteristic of the c.362C>T mutation identified in *PAX6* of the family members in the present study is that the mutation occurred at a hotspot for mutations. The location is consistent with this mutation’s presence for the first time in *PAX6* of patients with aniridia.

*PAX6* is located on chromosome 11p13. *PAX6* is divided into 14 exons that span 22 kb in length [40]. Human PAX6 is composed of two DNA-binding domains, the PD of 128 amino acids and the HD of 61 amino acids separated by a linker region of 79 amino acids, and is followed by a proline, serine, threonine-rich domain of 79 amino acids that have transcriptional trans-activation function [41].

The paired domain, which is encoded by exons 5–7 of PAX6, comprises two structurally distinct subdomains, the relatively conserved NH2 terminal (NTS) and the variable COOH terminal [42,43]. The NTS of the paired domain is highly conserved and plays an important role in contacting with the DNA. There is a helix-turn-helix unit, containing a β turn and three α helices (helix 1, 2, and 3, residues 23–35, 40–45, and 50–63, respectively) in the NTS; this helix-turn-helix unit makes critical contact in the sugar phosphate backbone, major groove, and minor groove. Among those residues, Arg38, Pro39, and Cys40 (Arg38 and Pro39 are in the turn between helices 1 and 2; Cys40 is part of helix 2) contact with the sugar phosphate backbone of the target DNA [44].

Interestingly, in our patients, the deletion mutation (c.112delC, p. Arg38GlyfsX16) affected residues (Arg38 to Val 53) involving the two amino acids (Arg38, Pro39, Cys40). Clinical data showed that although different patients had different symptoms, the patients have common and severe congenital anomalies in eye development, including the near absence of iris and congenital progressive cataract.

In summary, this study identified a *PAX6* mutation first reported in northern Chinese patients with aniridia. Our genetic analysis provides further examples of the haploinsufficiency of *PAX6* in aniridia. We also identified a novel *PAX6* mutation in a Chinese family with aniridia and congenital ptosis. This finding expands the mutation spectrum of *PAX6* and is valuable for genetic counseling and prenatal diagnosis in families where aniridia appears.

## Appendix 1. The clinical features of aniridia patients in the two Chinese families.

M: male; F: female; NA: not available; IOL: intraocular Lens; FC: finger counting. To access the data, click or select the words “Appendix 1.”
